# Nurse staffing configurations and sickness absence in English intensive care units: A longitudinal observational study

**DOI:** 10.1016/j.ijnsa.2025.100451

**Published:** 2025-11-10

**Authors:** Ezekwesiri Nwanosike, Chiara Dall’Ora, Peter Griffiths, Christina Saville, Thomas Monks, Natalie Pattison, Tolusha Dahanayake Yapa, SEISMIC-R study group

**Affiliations:** a University of Southampton, Faculty of Environmental and Life Sciences, Southampton, UK; b National Institute for Health Research Applied Research Collaboration (Wessex), Southampton, UK; c University of Exeter, Medical School, Southampton, UK; d University of Hertfordshire, School of Health and Social Work, Hatfield, UK; e East and North Hertfordshire NHS Trust, Stevenage, UK

**Keywords:** Covid-19, Critical care nursing, Nursing staff, Sickness absence, Skill mix

## Abstract

**Background:**

Staff wellbeing in intensive care units is essential for quality patient care, and nurse staffing configurations can impact nurse sickness absence. The COVID-19 pandemic imposed additional strain on nurses, potentially affecting sickness absence rates.

**Objective:**

To examine the association between registered nurse staffing levels, skill mix, and staff sickness absence in intensive care units spanning prepandemic (01/19–02/20), early pandemic (03/20–02/21), later pandemic (03/21–02/22), and post-pandemic (03/22–12/22).

**Design:**

Longitudinal retrospective study

**Setting(s):**

Three National Health Service hospital trusts in England

**Participants:**

Five intensive care units with 6916 sickness episodes from staffing data.

**Methods:**

We linked staffing data from electronic rostering systems. Variables included registered nurse hours per patient day, proportion of senior staff nurses with largely hands-on clinical experience, management presence, and sickness absence rates. Generalised linear mixed models analysed associations between staffing configurations in the previous 28 days and sickness absence.

**Results:**

The mean sickness absences rate was 2.4 %. When analysing all time periods collectively, an increase in registered nurse staffing by 1 standard deviation (SD) (11.0 h per patient day) was associated with a 5 % reduction in sickness episodes (incidence rate ratio [IRR]=0.95; 95 % confidence interval [CI] 0.90–0.99, *p* = 0.018); a 1 SD (15.1 %) increase in the proportion of senior nurse hours per patient day was associated with a 22 % reduction in sickness episodes (IRR=0.78; 95 % CI 0.71–0.86; *p* < 0.001). For management, the relationship exhibited a non-linear pattern, with both higher and lower levels of managerial presence, compared to the norm, being associated with increased sickness absence. The observed relationships changed over time, especially during later and post-pandemic periods. A 1 SD (11.7 h per patient day) increase in registered nurse staffing was associated with a 19 % reduction in sickness absence in the post-pandemic period (IRR 0.81; 95 % CI 0.69–0.95, *p* = 0.010). A 1 SD increase in proportion of senior nurse hours per patient day was associated with both reduced (IRR 0.60; 95 % CI 0.48–0.74, *p* < 0.001 later pandemic) and increased sickness absence (IRR 2.00; 95 % CI 1.31–3.05, *p* = 0.001 post pandemic).

**Conclusions:**

Sickness absence in intensive care units decreased with higher registered nurse staffing levels, although this relationship was most apparent post-pandemic. The presence of more senior registered nurses was generally associated with reduced sickness absence, although this relationship proved complex and varied across time periods. Pandemic conditions appear to have altered typical staffing-sickness patterns, with staff sickness being less influenced by workload during the acute pandemic phase.

**Social media abstract:**

Study of English intensive care units finds more senior nurses & higher staffing levels linked to reduced sickness absence—key for patient care quality!

**What is already known**

• Registered nurses’ well-being is crucial for quality patient care.
• Higher sickness absence rates are an indicator of poor nurse staff well-being.
• COVID-19 overstretched the registered nurse workforce in England.

**What this paper adds**

• When registered nurse staffing levels in the previous 28 days were higher, the rate of sickness absence was reduced.
• Senior nurse presence reduced subsequent sickness episodes
• The relationship between sickness absence and staffing levels changed during periods of the COVID-19 pandemic.

## 1 Background

Registered nurses (RNs) play a critical role in intensive care units (ICU) settings, and their availability and well-being are essential for maintaining patient safety and high standards of quality of care (Jun et al., 2021). High levels of sickness absence among RNs represent a significant operational challenge and are widely recognised as indicators of poor staff health, wellbeing (Kivimaki, 2003), and low job satisfaction (Kivimaki, 2003; Schaufeli et al., 2009). Sickness absence levels for registered nurses are typically high, incurring substantial costs to England’s National Health Service. Annual sickness absence among nursing staff costs over £443 million, with sickness absence episode averaging £2000 in temporary staffing and productivity losses (NHS-Digital, 2025; Turnbull, 2017). The consequences extend beyond financial costs to patient safety outcomes. Researchers have demonstrated that nurse understaffing and increased workload are associated with higher patient mortality rates and increased healthcare-associated infections.(Rae et al., 2021)

Stress and mental health-related conditions are the most reported reasons for sickness absence among RNs in England’s National Health Service, accounting for 25.2 % of all sickness absences (NHS-Digital, 2025). RN sickness absence was further impacted by the COVID-19 pandemic, especially in the ICUs (Stilwell, 2024). The surge in the demand for critical care during the COVID-19 pandemic, amid vulnerabilities in health systems worldwide, imposed additional strain on already overworked nurses in the ICU (Stilwell, 2024). Notably, the onset of the pandemic brought about changes in sickness absence policies for health staff (e.g., duration of absence due to isolation and testing protocols), ultimately affecting their sickness absence rates and availability (Endacott et al., 2022; Pattison, 2021; Stilwell, 2024).

There is also little evidence on the predictors of sickness absence in nursing (Brborović et al., 2017), and studies in the ICU have focused mainly on nurse wellbeing rather than on sickness absence specifically (Stewart, Bench and Malone, 2024). In addition, many existing studies relied on surveys where employees self-reported working conditions, which were then linked to objective sickness records, although the quality of these varied (Brborović et al., 2017; Helgesson et al., 2020). Researchers using routinely collected data extracted from hospital systems highlighted associations between nursing shift patterns and absenteeism, with higher rates of sickness absence found when nurses worked long shifts of 12 h or more (Dall’Ora et al., 2019; Rodriguez Santana et al., 2020) and higher proportions of night shifts (Dall’Ora et al., 2020). One recent study found that RN understaffing and lower RN skill mix are associated with higher RN sickness absence in inpatient settings. While ICUs were part of the sample, it was not possible to extract ICU-specific results (Dall’ora et al., 2024).

We are not aware of any studies that examined the relationship between RN staffing and sickness absence in the ICUs, especially in the context of the COVID-19 pandemic. We aimed to measure the association between RN staffing levels, skill mix, and sickness absence in English ICUs over four years, including the pandemic period.

## 2 Methods

This was a longitudinal retrospective study using staffing data from the electronic roster systems of three acute National Health Service hospital trusts (five ICUs from the Midlands to London with bed capacities ranging from 20 to 80) in England. The RECORD reporting guideline was followed (Benchimol et al., 2015). Patient data were provided by the Intensive Care National Audit and Research Centre (ICNARC) via their Data Access Advisory Group (201,401). Given our interest in nurse staffing configurations in the context of the COVID-19 pandemic, we extracted data from January 2019 to December 2022.

### 2.1 Data and variables

The staffing data included worked shifts of nurses in the ICU, which we converted into worked hours per day shift interval (7 am to 6.59 pm) and night shift interval (7 pm to 6.59 am) to align approximately with typical nurse shifts. This was matched to records with sickness absences and aggregated at the ward-day level.

The United Kingdom (UK) nursing workforce is organised according to the National Health Service (NHS) NHS Agenda for Change banding system (NHS, 2025):

Band 5 RNs: Newly qualified registered nurses or in the early stages of their career. This is the entry-level position for registered nurses within the NHS Agenda for Change pay structure.

Band 6 RNs: Experienced registered nurses with advanced clinical skills

Band 7+ RNs: Senior nurses with managerial responsibilities who may provide direct patient care when necessary but primarily focus on leadership, coordination, and clinical oversight

We classified junior RNs in Band 5 and senior RNs in Band 6 to reflect skill, competence, and seniority (Royal College of Nursing, 2019). Nurse managers (band 7 and above) were grouped separately, as these nurses do not routinely deliver direct care at the bedside. As a group, they have a limited and uncertain contribution to direct patient care.

Meanwhile, the patient records and staffing data (worked shifts) were linked to calculate the RN hours per patient day(s): the number of staff hours per day divided by patient days. The data available was from March 2019 to December 31, 2022. This accounts for changes in ward staffing in periods before, during, and after the COVID pandemic: *prepandemic* (01/19–02/20), *early pandemic* (03/20–02/21), *later pandemic* (03/21–02/22), and *post-pandemic* (03/22–12/22).

Being the best proxy for direct care hours (hands-on patient care), the absolute hours per patient day value for Bands 5 and 6 RNs (RN staff nurse hours per patient day) in the ward was used as the main indicator for staffing. We captured skill mix by including the proportion of Band 6 RNs. We analysed the proportion of Band 6 RNs separately because this measure captured skill mix quality rather than just quantity. While Band 5 + Band 6 h per patient day represented total direct care nursing hours, the proportion of Band 6 nurses reflected the level of experience within that total. This distinction is crucial because experienced ICU nurses can manage more complex cases, mentor junior staff, and potentially prevent situations that might lead to staff stress and subsequent sickness absence. We also included hours per patient day from Band 7+ RNs (reflecting staffing by managers who are normally not providing direct care). The averages for these continuous variables were determined, and the 28-day lookback period was used; a sufficient time window to capture the impact of staffing on sickness absence (Ropponen et al., 2019).

Our outcome measure was the number of sickness episodes on each ward day, and we adjusted for the total worked shifts in our models. The rate of sickness episodes was calculated as sickness episodes/ (sickness episodes + worked shifts) x 100. For sensitivity analysis (see Supplementary Material), we explored the effect of low staffing in the ward (i.e., RN hours per patient day <24 h of care or a 1:1 nurse-to-patient ratio), expressed as the proportion of days of understaffing within the 28 days leading up to the day of absence.

In the UK, ICU nursing follows a 1:1 nurse-to-patient ratio for critically ill patients, as recommended by national guidelines (Faculty of Intensive Care Medicine and Intensive Care Society, 2022). The 24 h per patient day threshold used in our analysis represents this 1:1 standard (24 h of nursing care per patient per day).

For international readers, our findings should be interpreted within this context—the 'understaffing' threshold of <24 h per patient day represents deviation from UK standards and may not directly translate to healthcare systems with different baseline staffing ratios. However, the relative relationships between staffing levels, skill mix, and sickness absence are likely to be relevant across different healthcare contexts. It is important to note that hours per patient day values above 24 do not indicate "overstaffing" but reflect the operational realities of delivering safe ICU care. Higher staffing levels occur when: (1) patients require enhanced observation or 2:1 nursing ratios due to clinical instability and complex procedures; (2) units must maintain adequate staffing to manage unpredictable admission patterns and emergency situations; (3) staff must be present before anticipated admissions, particularly for elective post-surgical cases where precise timing cannot be guaranteed; and (4) sufficient flexibility is needed to respond to varying patient dependency levels throughout shifts.

### 2.2 Data management

Data preparation and analyses were carried out using R version 4.3.1 (R Project for Statistical Computing) with RStudio (Posit). For data wrangling and summary statistics, we used *tidyverse* 2.0.0 (Wickam, 2023) and *GTsummary* 1.7.2 packages (Sjoberg et al., 2024), respectively. The numerous job titles identifying the nursing staff in the roster dataset were categorised into major staff groups (mentioned above) with the open-source *OpenRefine* software (version 3.7.7) and regular expressions (regex) in R. The irrelevant job titles (e.g., nurse educators) were excluded. For analysis of the staffing-absences dataset, we used the Linear mixed effect modelling (*lme4)* package 1.1–34 (Bates et al., 2023). The significance level for statistical inference was set at *p* < 0.05. Records in the linked data without look-back periods were excluded, as were records with missing values.

### 2.3 Statistical analysis

We used the generalised linear mixed models (GLMM) framework to model the Poisson distribution of the count of sickness episodes (Kivimäki et al., 2004). We then added the staffing variables (i.e., RN staff nurse hours per patient day, proportion of experienced (Band 6) RN staff nurse and RN Band 7+ hours per patient day (management presence)) as fixed effects. We tested for non-linear effects by adding cubic and quadratic terms and retained these where model fit was improved (Akaike Information Criterion [AIC] / Bayesian Information Criterion [BIC] reduced. We standardised the staffing variables by scaling (standardisation), allowing us to interpret effects in terms of 1 standard deviation (SD) change in exposure variables. All models had ICU as random effect to ensure all data were clustered within ICUs, as local variation in policy and practice might influence sickness absence levels. The combined multivariable model included all the staffing variables. Relative model quality (fit) was evaluated based on AIC with a preference for models with minimal values. To assess how associations changed over time, we fitted the models into subgroups of the data corresponding to annual periods from prepandemic to post-pandemic.

### 2.4 Approvals

Ethics approval for this study (NIHR funder reference: 135,168) was obtained from HRA (IRAS 316,667 and REC 23/SW/0028) and the University of Southampton Ethics Committee (ERGO reference: 80,440). A waiver of informed consent was applicable because all the data supplied were anonymous (NHS Health Research Authority, 2022).

## 3 Results

**Fig. 1:**
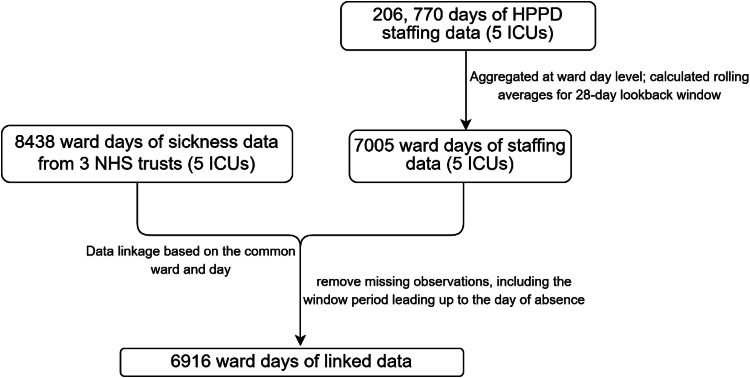
Flow diagram showing how analyses dataset was derived. Notes: HPPD = Hours per patient day.

The linked dataset comprised five ICUs in three NHS trusts with 6916 ward days of linked data (see Fig. 1). Across ICUs, there was an overall daily mean absence rate of 2.4 % (Table 1). The overall mean RN Staff nurse hours per patient day was 32.5 (SD=11.0), indicating that study ICUs maintained staffing above the UK's recommended 1:1 standard on average. This reflects the intensive nature of UK ICU care, where continuous 1:1 nursing is supplemented by additional nurses based on patient acuity and dependency levels. Our data do not distinguish between those delivering direct care and those in supernumerary roles, such as nurse leaders.

**Table 1:** Staffing levels and staff mix.

	ALL PERIODS (*n* = 6916)				prepandemic (*n* = 1692)				early pandemic (*n* = 1935)				later pandemic (*n* = 1806)				post-pandemic (*n* = 1483)			
VARIABLE	Mean	(SD)	Median	(IQR)	Mean	(SD)	Median	(IQR)	Mean	(SD)	Median	(IQR)	Mean	(SD)	Median	(IQR)	Mean	(SD)	Median	(IQR)
Absence rate (sickness episodes)	2.44	(5.17)	1.2	(3.2)	1.8	(2.6)	0.0	(2.9)	3.1	(7.1)	1.4	(3.3)	2.6	(6.0)	1.5	(3.4)	2.2	(2.5)	1.6	(3.3)
RN staff nurse (Band 5 & 6) hours per patient day (HPPD)	32.53	(11.03)	28.5	(19.0)	28.7	(8.0)	25.9	(10.4)	31.9	(9.9)	29.1	(12.8)	36.1	(12.7)	31.1	(23.1)	33.4	(11.7)	26.9	(21.5)
%Days of low staffing (<24 HPPD)	29.00	(35.02)	10.0	(60.0)	39.7	(37.5)	30.0	(70.0)	25.8	(30.8)	10.0	(50.0)	22.1	(34.7)	0.0	(20.0)	29.2	(34.9)	10.0	(60.0)
% of Band 6 staff nurse HPPD ( %)	31.52	(15.14)	23.1	(28.7)	31.5	(14.5)	26.0	(28.9)	33.6	(13.9)	30.0	(23.7)	32.0	(17.3)	21.3	(32.1)	28.3	(14.1)	19.2	(28.7)
RN Band 7+ HPPD	2.75	(1.52)	2.3	(2.3)	2.1	(1.5)	1.6	(0.8)	3.2	(1.5)	2.9	(1.9)	2.8	(1.4)	2.7	(2.6)	2.9	(1.5)	2.9	(2.4)

Absence rate = sickness episodes/(sickness episodes + worked shifts) x 100 (i.e., expressed as percent).

Notes: HPPD = Hours Per Patient Day; SD = Standard Deviation; IQR = Inter Quartile Range; *n* = number; RN = Registered Nurse.

While this may appear as 'overstaffing' compared to countries using 2:1 patient-to-nurse ratios, it aligns with UK ICU guidelines for critically ill patient care. Other staff mix factors (e.g., proportion of experienced Band 6 RN staff nurse) showed a similar trend, as shown in Table 1. RN Staff nurse hours per patient day (hours per patient day) peaked at a mean of 36.1 (SD=12.7) during the later pandemic, dipping to its lowest during the post-pandemic period (26.9 [SD=21.5]). Low RN staffing was most common during the prepandemic period and least common during the later pandemic period. The Band 7 RN (managers) hours per patient day was highest during the early pandemic phase and lowest during the prepandemic phase. The sickness absence rate was highest during the early pandemic period (3.1 % [SD=7.1]) and lowest during the prepandemic phase.

**Fig. 2:**
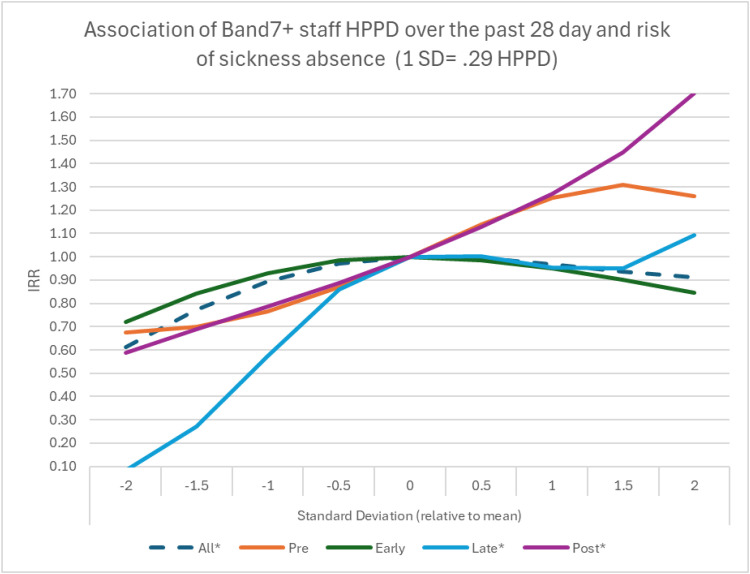
Nonlinear effect of Band 7+ on sickness absence. Notes: HPPD = Hours per patient day; SD = Standard Deviation.

Overall, an increase in RN nurse staffing by 1 SD (11.0 h per patient day) is associated with a 5 % decrease in sickness episodes (incidence rate ratio [IRR]=0.95; 95 % CI: 0.90–0.99, *p* = 0.018), while increase in the proportion of experienced (Band 6) RN nurse staffing by 1 SD (15.1 percentage points) was associated with a 22 % decrease in sickness episodes (IRR=0.78; 95 % CI: 0.71–0.86, *p* < 0.001). We found evidence of a non-linear association between management presence in the previous 28 days and sickness absence. Because nonlinear effects are difficult to convey based on point estimates only, we produced graphical representations from the IRRs of squared and cubic terms. Overall, a higher presence of management is associated with increased sickness absence (significant in 2/4 time periods), a relationship clearly not present early pandemic (see Table 2 and Fig. 2).

**Table 2:** Models for the association of staffing and sickness absence including all staffing factors.

Time period	Variable	IRR	95 % CI	p.value
All*	RN Staff nurse HPPD	0.95	0.90, 0.99	0.018
	Proportion Band 6	0.78	0.71, 0.86	<0.001
	RN Band 7+ HPPD	1.02	0.96, 1.08	0.6
	RN Band 7+ HPPD squared**	0.93	0.88, 0.97	0.002
	RN Band 7+ HPPD cubic**	1.02	1.00, 1.04	0.014
	*(AIC)*	*18,071.2*		
prepandemic	RN Staff nurse HPPD	1.08	0.81, 1.43	0.6
	Proportion Band 6	1.07	0.73, 1.57	0.7
	RN Band 7+ HPPD	1.32	0.72, 2.43	0.4
	RN Band 7+ HPPD squared	0.98	0.84, 1.15	0.8
	RN Band 7+ HPPD cubic	0.97	0.83, 1.12	0.6
	*(AIC)*	*3493.1*		
Early pandemic	RN Staff nurse HPPD	1.04	0.97, 1.11	0.2
	Proportion Band 6	1.10	0.95, 1.28	0.2
	RN Band 7+ HPPD	1.00	0.91, 1.10	>0.9
	RN Band 7+ HPPD squared	0.94	0.85, 1.04	0.2
	RN Band 7+ HPPD cubic	1.01	0.98, 1.04	0.4
	*(AIC)*	*5466.1*		
Later pandemic	RN Staff nurse HPPD	0.93	0.80, 1.08	0.3
	Proportion Band 6	0.60	0.48, 0.74	<0.001
	RN Band 7+ HPPD	1.13	0.94, 1.36	0.2
	RN Band 7+ HPPD squared	0.74	0.64, 0.86	<0.001
	RN Band 7+ HPPD cubic	1.14	1.03, 1.25	0.008
	*(AIC)*	*4848.0*		
post-pandemic	RN Staff nurse HPPD	0.81	0.69, 0.95	0.010
	Proportion Band 6	2.00	1.31, 3.05	0.001
	RN Band 7+ HPPD	1.27	1.00, 1.62	0.048
	RN Band 7+ HPPD squared	0.92	0.77, 1.10	0.4
	RN Band 7+ HPPD cubic	0.98	0.91, 1.06	0.7
	*(AIC)*	*3805.9*		

*All controlled for year of admission (pandemic period) |Effect size scaled to SD unit.

**Adding polynomial terms (squared i.e., ^2 and cubic i.e., ^3, respectively).

Notes: HPPD = Hours Per Patient Day; IRR = Incidence Rate Ratio; 95 % CI = 95 % Confidence Interval; AIC = Akaike Information Criteria; RN = Registered Nurse.

There were some differences across the periods we explored. For example, a higher proportion of experienced (Band 6) RN staff nurses during the later pandemic became significantly associated with lower sickness absence, but in the post-pandemic period, the association changed in the opposite direction. In other words, during the later pandemic period, an increase in proportion of experienced (Band 6) RN nurse staffing by 1 SD (17.3 h per patient day) was associated with a 40 % reduction in sickness episodes (IRR=0.60; 95 % CI: 0.48–0.74, *p* < 0.001), whereas, during the post-pandemic period, It was associated with a two-fold increase in sickness episodes (IRR=2.00; 95 % CI: 1.31–3.05, *p* = 0.001) respectively (Table 2).

## 4 Discussion

This is the first study to measure the association between RN staffing configurations in the ICU and their risk of sickness absence. Overall, more RN hours per patient day was associated with lower sickness. When there were higher proportions of more experienced staff nurses, we observed lower sickness absence levels. However, results varied by time period, and there was confusing and contradictory evidence about the association between having more experienced staff nurses and more senior staff.

The absence rate peaked in the early pandemic, likely due to isolation policies at the time, which required anyone contracting COVID-19 to self-isolate for 14 days. While our findings show decreased sickness absence in later pandemic periods, this reduction may be partially attributed to COVID-19 vaccination programmes that became widely available to healthcare workers from early 2021, potentially preventing some illness episodes and reducing mandatory isolation periods. However, this does not diminish the evidence that staff remained overstretched and overburdened throughout the pandemic, as evidenced by the sustained higher absence rates compared to prepandemic levels and the altered relationships between staffing configurations and sickness patterns.

The protective effect of RN staff nurses, overall, aligns with the findings of Dall’Ora et al. (2025), although their study was not ICU-specific. The variation in effect across COVID periods suggests the associations observed during the COVID-19 pandemic may be confounded by factors, such as government-mandated self-isolation policies and redeployment decisions that varied across ICUs, leading non-ICU qualified nursing staff to provide care in high-pressure critical care settings (Pattison, 2021).

Higher proportions of experienced (Band 6, ICU qualified) RN staff nurses positively influenced sickness absence during the later pandemic, although this association reversed in the post-pandemic period. Band 6, experienced ICU nurses, were heavily utilised in the early pandemic but less so in the later pandemic. Modelling Band 7+ nurse levels (management presence) suggests non-linear associations overall, driven mainly by the atypical period. Any protective effect could be due to Band 7+ nurses (management presence) acting as motivators, an additional clinical expertise resource, and fostering positive team morale in the ICU. However, more sickness absences when management hours were higher in the previous 28 days could reflect their deployment in response to shortfalls in experienced nurse staff due to sickness absence.

Despite these hypothesised mechanisms, the COVID-19 periods were marked by numerous factors that may have confounded the associations we identified. These include the reassignment of nursing roles (with more support staff deployed to minimise shortfalls), the creation of new wards, changes in hospital layout, and patient-related factors such as variations in acuity/case mix (Endacott et al., 2022; Pattison, 2021).

## 5 Limitations

In addition to the inability to control for the aforementioned confounding mechanisms, our study has several limitations. Firstly, we relied on a limited sample of three NHS trusts, which may not be representative of the entire UK, although these sites are geographically dispersed and vary in size and the populations they serve. Secondly, we acknowledge that some effect sizes are modest with confidence intervals approaching unity, suggesting these associations may be sensitive to additional confounding variables not captured in our models. The complexity of factors influencing sickness absence in ICUs — including individual nurse characteristics, working conditions, organisational culture, and external stressors — likely contribute to the relatively small effect sizes observed. Future research incorporating more granular individual-level data and organisational factors would strengthen causal inference. Despite these limitations, we have provided important evidence for the relationship between staffing configurations and nurse wellbeing in critical care settings. Thirdly, as we could not link individual staff records over time, our analysis was conducted at the ward day level; we cannot be sure that the individuals who were absent due to sickness had experienced low staffing over the past 28 days, although the length of the look back period means it is likely to be a good indicator of staffing levels experienced by staff taking sick leave on most occasions.

## 6 Conclusions

The COVID-19 pandemic significantly altered RN deployment in English ICUs. We observed considerable variation in associations between RN staffing configurations and their sickness absence across different COVID-19 periods. Protective factors in the late pandemic, such as higher levels of experienced Band 6 RN staff nurses, did not have similar effects post-pandemic; non-linearities in associations with management (Band 7+ RN) were noted (overall pattern with management presence was inconsistent, suggesting cautious interpretation of beneficial or harmful effects). While further research could provide more insights into RN deployment patterns, such as the impact of ICU-qualified nurses on sickness absence, we have contributed to the evidence that variations in RN staffing configurations affect nurses' health and well-being. Given the substantial burden of sickness absence, investing in adequate levels of RNs is likely to alleviate pressures on health systems, especially in the event of another pandemic.

## Data availability

Data-sharing agreements with the organisations that provided data prevent us from sharing data with third parties.

## Disclaimer

This paper presents independent research commissioned by the NIHR. The views expressed are those of the author(s) and not necessarily those of the NIHR or the Department of Health and Social Care.

## Declaration of competing interest

All authors declare funding from National Institute for Health and Care Research (England) (NIHR) as Payment to institution – grant funding to undertake the research. PG, CS and CDO declare funding from NHS England as Payment to institution for undertaking assessments of research relating to safe staffing for national guidance. NP received funding from the RCN foundation and acted as Chair of Trustees and Chair of National Outreach Forum (registered UK charity 1181,360): unpaid.

## Funding

This project was funded by the National Institute for Health and Care Research (NIHR) Health Services and Delivery Research Programme (award ID: NIHR135168). The views expressed are those of the author(s) and not necessarily those of the NIHR or the Department of Health and Social Care

## CRediT authorship contribution statement

**Ezekwesiri Nwanosike:** Writing – review & editing, Writing – original draft, Visualization, Formal analysis, Data curation. **Chiara Dall’Ora:** Writing – review & editing, Writing – original draft, Supervision, Project administration, Methodology, Funding acquisition, Conceptualization. **Peter Griffiths:** Writing – review & editing, Writing – original draft, Supervision, Methodology, Funding acquisition, Conceptualization. **Christina Saville:** Writing – review & editing, Writing – original draft, Supervision, Project administration, Methodology, Funding acquisition. **Thomas Monks:** Writing – review & editing, Methodology, Funding acquisition. **Natalie Pattison:** Writing – review & editing, Supervision, Funding acquisition. **Tolusha Dahanayake Yapa:** Writing – review & editing, Validation, Formal analysis.

## Declaration of competing interest

The authors declare the following financial interests/personal relationships which may be considered as potential competing interests:

Ezekwesiri Nwanosike reports financial support was provided by National Institute for Health and Care Research. If there are other authors, they declare that they have no known competing financial interests or personal relationships that could have appeared to influence the work reported in this paper.

## Appendix Supplementary materials

Image, application 1
