# Supplementary material for: Nurse staffing configurations and sickness absence in English intensive care units: A longitudinal observational study
Source: Int J Nurs Stud Adv. 2025 Nov 10;9:100451. doi: 10.1016/j.ijnsa.2025.100451 (PMC12664349; doi:10.1016/j.ijnsa.2025.100451)
Supplement: Supplementary file 1 [file mmc1.docx]

*Supplementary:* *Table 3:Models for the association of staffing and sickness absence including – single staff mix factors*

|  | Variable |  | IRR | p.value | 95% CI | AIC |
| --- | --- | --- | --- | --- | --- | --- |
| All periods | RN Staff Nurse (SN) HPPD |  | 0.94 | 0.002 | 0.90, 0.98 | 21538.8 |
| * | *Low (<24 RN SN HPPD)* |  | 1.03 | 0.2 | 0.98, 1.07 | 18101.3 |
|  | Proportion Band 6 SN |  | 0.81 | <0.001 | 0.74, 0.89 | 18076.1 |
|  | RN Band 7+ HPPD |  | 1.02 | 0.5 | 0.97, 1.07 | 18094.6 |

* = alternative measure of staffing (understaffing) scaled by SD

*Supplementary Table 4: Models for the association of low staffing and sickness absence including all staff mix factors*

| Time period | Variable | IRR | 95% CI | p.value |
| --- | --- | --- | --- | --- |
| All* | <24 RN SN HPPD | 1.02 | 0.98, 1.07 | 0.3 |
|  | Proportion Band 6 SN | 0.77 | 0.70, 0.85 | <0.001 |
|  | RN Band 7+ HPPD | 0.99 | 0.94, 1.05 | 0.7 |
|  | RN Band 7+ HPPD ^2** | 0.92 | 0.88, 0.97 | 0.001 |
|  | RN Band 7+ HPPD ^3** | 1.02 | 1.01, 1.04 | 0.008 |
|  | *(AIC)* | *18075.8* |  |  |
| prepandemic | <24 RN SN HPPD | 1.08 | 0.81, 1.43 | 0.6 |
|  | Proportion Band 6 SN | 1.07 | 0.73, 1.57 | 0.7 |
|  | RN Band 7+ HPPD | 1.32 | 0.72, 2.43 | 0.4 |
|  | RN Band 7+ HPPD ^2** | 0.98 | 0.84, 1.15 | 0.8 |
|  | RN Band 7+ HPPD ^3** | 0.97 | 0.83, 1.12 | 0.6 |
|  | *(AIC)* | *3493.1* |  |  |
| Early pandemic | <24 RN SN HPPD | 1.08 | 1.00, 1.17 | 0.049 |
|  | Proportion Band 6 SN | 1.12 | 0.96, 1.30 | 0.2 |
|  | RN Band 7+ HPPD | 1.05 | 0.96, 1.15 | 0.3 |
|  | RN Band 7+ HPPD ^2** | 0.94 | 0.85, 1.04 | 0.2 |
|  | RN Band 7+ HPPD ^3** | 1.01 | 0.98, 1.04 | 0.4 |
|  | *(AIC)* | *5463.7* |  |  |
| Later pandemic | <24 RN SN HPPD | 1.02 | 0.91, 1.15 | 0.7 |
|  | Proportion Band 6 SN | 0.60 | 0.48, 0.74 | <0.001 |
|  | RN Band 7+ HPPD | 1.12 | 0.93, 1.35 | 0.2 |
|  | RN Band 7+ HPPD ^2** | 0.74 | 0.63, 0.88 | <0.001 |
|  | RN Band 7+ HPPD ^3** | 1.13 | 1.03, 1.25 | 0.013 |
|  | *(AIC)* | *4848.8* |  |  |
| post-pandemic | <24 RN SN HPPD | 1.38 | 1.24, 1.53 | <0.001 |
|  | Proportion Band 6 SN | 2.21 | 1.46, 3.34 | <0.001 |
|  | RN Band 7+ HPPD | 1.47 | 1.16, 1.88 | 0.002 |
|  | RN Band 7+ HPPD ^2** | 0.87 | 0.73, 1.03 | 0.11 |
|  | RN Band 7+ HPPD ^3** | 0.98 | 0.91, 1.06 | 0.6 |
|  | *(AIC)* | *3774.4* |  |  |

Sensitivity analysis based on low staffing showed broadly similar (expected) results, especially in terms of nonlinear effects | **All* controlled for year of admission (pandemic period) |Effect size scaled to SD unit
